# Impact of oropharyngeal dysphagia on healthcare cost and length of stay in hospital: a systematic review

**DOI:** 10.1186/s12913-018-3376-3

**Published:** 2018-08-02

**Authors:** Stacie Attrill, Sarahlouise White, Joanne Murray, Sue Hammond, Sebastian Doeltgen

**Affiliations:** 1 Speech Pathology, College of Nursing and Health Sciences, Flinders University, GPO Box 2100, Adelaide, SA 5000 Australia; 2 Library Services, Flinders University, Adelaide, Australia; 3 Swallowing Neurorehabilitation Research Laboratory, College of Nursing and Health Sciences, Flinders University, Adelaide, Australia

**Keywords:** Meta-analysis, Financial, Swallowing, Costs, Expenditure

## Abstract

**Background:**

Healthcare systems internationally are under an ever-increasing demand for services that must be delivered in an efficient, effective and affordable manner. Several patient-related and organisational factors influence health-care expenditure and utilisation, including oropharyngeal dysphagia. Here, we present a systematic review of the literature and meta-analyses investigating how oropharyngeal dysphagia influences healthcare utilisation through length of stay (LOS) and cost.

**Methods:**

Using a standardised approach, eight databases were systematically searched for relevant articles reporting on oropharyngeal dysphagia attributable inpatient LOS and healthcare costs through June 2016. Study methodologies were critically appraised and where appropriate, extracted LOS data were analysed in an overall summary statistic.

**Results:**

Eleven studies reported on cost data, and 23 studies were included reporting on LOS data. Descriptively, the presence of dysphagia added 40.36% to health care costs across studies. Meta-analysis of all-cause admission data from 13 cohort studies revealed an increased LOS of 2.99 days (95% CI, 2.7, 3.3). A subgroup analysis revealed that admission for stroke resulted in higher and more variable LOS of 4.73 days (95% CI, 2.7, 7.2). Presence of dysphagia across all causes was also statistically significantly different regardless of geographical location: Europe (8.42 days; 95% CI, 4.3; 12.5), North America (3.91 days; 95% CI, 3.3, 4.5). No studies included in meta-analysis were conducted in Asia.

**Conclusions:**

This systematic review demonstrated that the presence of oropharyngeal dysphagia significantly increases healthcare utilisation and cost, highlighting the need to recognise oropharyngeal dysphagia as an important contributor to pressure on healthcare systems.

**Electronic supplementary material:**

The online version of this article (10.1186/s12913-018-3376-3) contains supplementary material, which is available to authorized users.

## Background

Healthcare systems internationally are under an ever increasing demand for services that must be delivered in an effective and affordable manner. Simultaneously, there is increasing pressure to optimise patient outcomes and meet clinical and operational benchmarks that ensure service quality [1–3]. As healthcare expenditures have increased, research investigating affordability, cost-containment policies and features of healthcare utilisation has become more prominent in the literature. This has identified several patient-related and organisational factors that influence healthcare expenditure [3]. Oropharyngeal dysphagia is one such patient-related symptom that is common to several complex medical conditions, and also influences organisational factors related to hospital procedures, availability and training of staff and the application of clinical pathways [4, 5]. However, there has been no systematic investigation of how oropharyngeal dysphagia influences healthcare utilisation and cost.

The contributors to expenditure and utilisation in the provision of healthcare are complex and multilayered, and consensus is lacking about definitions for these, or measures to capture them accurately. Previous studies have often measured cost of care through patient-related factors including Diagnosis-Related Group (DRG) models, patient acuity and hospital length of stay (LOS) [1–3]. These measures have been criticised as they do not incorporate organisational factors including the intensity and coordination of patient care, or account for underreported symptoms such as oropharyngeal dysphagia [2, 3, 6]. Measuring LOS as a proxy for healthcare expenditure is particularly problematic, as the intensity of patient care has been shown to increase as LOS is shortened [3]. However, identifying how patient factors contribute to hospital related expenditure remains important to plan for demand and activity organisation, and LOS continues to provide utilisation information of relevance for hospital bed management and capacity planning [1].

There are several symptoms common to a range of conditions that are known to increase health care utilisation through patient and organisational factors. For example, malnutrition [7], severe anemia [8] and delirium [9] are each associated with increased LOS in certain populations. One such symptom that has not been systematically investigated in relation to its influence on LOS or cost is oropharyngeal dysphagia, or swallowing impairment. The presence of dysphagia is associated with reduced quality of life [10], malnutrition [11], dehydration [12]^,^ and poor healthcare outcomes [13] including aspiration pneumonia, which is the second leading cause of death in the elderly [14]. Oropharyngeal dysphagia is a direct and critical symptom of a range of conditions known to contribute to high healthcare expenditure, including stroke [15], traumatic brain injury [16] and head and neck cancer [17]. In many of these conditions, the presence of dysphagia predicts greater severity of disease and poorer health outcomes, which are also correlated with greater utilisation of healthcare [3]. In studies related to these conditions, oropharyngeal dysphagia is often a secondary contextual measure that is not commonly reported as a primary outcome. Therefore, data about healthcare cost and utilisation related to oropharyngeal dysphagia is difficult to access, inconsistently measured and reported, and subject to variable research foci and methodologies.

However, a recent study has quantified the impact of dysphagia on the cost of healthcare and LOS in the United States of America (USA) by analysing International Classification of Disease (ICD)-9 codes from national inpatient discharge data. The extracted data did not discern patients with oropharyngeal dysphagia from those with esophageal dysphagia, instead treating these as a single condition. Overall, inpatients with dysphagia were noted to increase LOS by 8.8 days and cost 42% more per admission than patients without dysphagia [18]. However, the assessment and management of oropharyngeal and esophageal dysphagia differ substantially due to their aetiologies and the use of discrete pharmacological and surgical interventions for esophageal dysphagia. Furthermore, organisational factors related to oropharyngeal dysphagia include complex assessment and management guidelines, multidisciplinary support needs, ongoing reliance on modified diet or supplementary feeding and increased likelihood of discharge into a skilled nursing facility [15] that are under-recognised contributors to increased direct and indirect healthcare utilisation. As such, the financial consequences of oropharyngeal dysphagia on any healthcare system are likely to be substantial and require systematic evaluation that is distinct from esophageal dysphagia.

Quantifying the impact of oropharyngeal dysphagia on healthcare utilisation is critical to enable managers, clinicians and patients to advocate for efficient and evidence-based strategies to manage, or prevent its detrimental sequelae and may facilitate appropriate allocation of resources within healthcare systems [3]. To provide more information to quantify the impact of oropharyngeal dysphagia, the purpose of the current study was to systematically review findings of studies on health care expenditure associated with oropharyngeal dysphagia through the parallel review of studies that reported on cost and hospital LOS. In this review, reported monetary costs and LOS were variables considered to proxy for healthcare expenditure as these may be directly translated into different healthcare systems internationally and provide information about hospital utilisation to inform contemporary hospital resourcing.

## Methods

This systematic review was conducted using a standardised methodology and critical appraisal tools from the Joanna Briggs Institute [19]. Study identification was reported according to the Preferred Reporting Items for Systematic Reviews and Meta-analysis (PRISMA) statement [20] and meta-analysis was conducted using The Cochrane Collaboration RevMan 5.3 software [21]. The presence of heterogeneity in the meta-analyses was determined using the standard Chi-square test. The degree of heterogeneity was assessed using I^2^.

### Objectives

The review sought to synthesise the best available evidence in relation to inpatient care related LOS and care setting financial costs for patients with oropharyngeal dysphagia, from the viewpoints of both patients and healthcare providers. More specifically, the review questions were:

1. What is the inpatient admission related expenditure, in monetary terms, of patients with oropharyngeal dysphagia, compared with their etiology-matched peers without dysphagia?
2. What is the impact on the length of inpatient care stay, of patients with oropharyngeal dysphagia, compared with their etiology-matched peers without dysphagia?

### Definitions

The following definitions were utilised for this review:

- Cost: reference to financial cost or economic impact in any care setting.
- Length of stay (LOS): mean or median number of patient days between a formal admission to, and a formal separation from a hospital care environment [22].
- Dysphagia: reference to a patient group with impaired oral and/or pharyngeal swallowing (oropharyngeal dysphagia).

### Inclusion criteria

#### Types of participants

This review considered studies of adult patients, of any ethnic background, with or without co-morbidities, admitted to an inpatient care setting with any diagnosis. Those studies that included patients with swallowing disorder or oropharyngeal dysphagia, and reference to either LOS in any inpatient hospital facility (acute or rehabilitation hospital setting) OR reference to financial or economic cost in any care setting were considered for inclusion.

Studies were excluded if they sampled pediatric patients; investigated patients with esophageal dysphagia; or where LOS or cost related to, or were not corrected for, tracheostomy or a surgical intervention (e.g. pharyngeal pouch repair) rather than the oropharyngeal dysphagia itself. Papers that examined cost or LOS of an intervention and/or tools for managing oropharyngeal dysphagia (e.g. costs of dysphagia screening), rather than generalised differences in cost or LOS for patient groups with and without oropharyngeal dysphagia were also excluded.

#### Type of outcomes

Studies were considered to address Review question 1 if they referred to financial or economic costs of oropharyngeal dysphagia in any care setting, where these costs were reported with a monetary value in any currency for comparative patient groups with and without dysphagia.

Studies were considered for Review question 2 if they reported mean or median length of inpatient stay in any hospital facility for patient groups with and without oropharyngeal dysphagia.

#### Types of studies

This review considered any primary research studies utilising quantitative study designs that met the inclusion criteria, including, but not limited to: randomised controlled trials (RCTs), cohort studies, cross-sectional studies and case controlled studies.

#### Search strategy

The search strategy was developed with a medical librarian (SH) using subject headings and text words relevant to dysphagia, costs and LOS. The search strategy was tested and finalised in Medline (Ovid) and then translated into the following databases using the equivalent subject headings, all text words, and with syntax adapted accordingly: PubMed (non-indexed subset only), Scopus, CINAHL (Ebsco), PsycInfo (Ovid), Cochrane Library, Web of Science, and ProQuest. The search was limited to English language publications. No date limits were applied to the search, as it was anticipated that few studies would include comparable groups with and without oropharyngeal dysphagia. The searches were run during February 2016, and the results were exported and de-duplicated in Endnote X8 bibliographic software (http://endnote.com/). The full search strategies for each database are detailed in Additional file 1. The reference lists of all identified relevant studies and articles were hand searched for additional studies.

In an attempt to address publication bias, a grey literature search was undertaken to identify relevant unpublished literature. The internet was searched via Google, limiting to PDFs and using search terms dysphagia and cost/s. The first 200 results were scanned for relevant studies. The following websites were also searched: Australian Institute of Health and Welfare (AIHW), Opendoar and Trove.

**Fig. 1:**
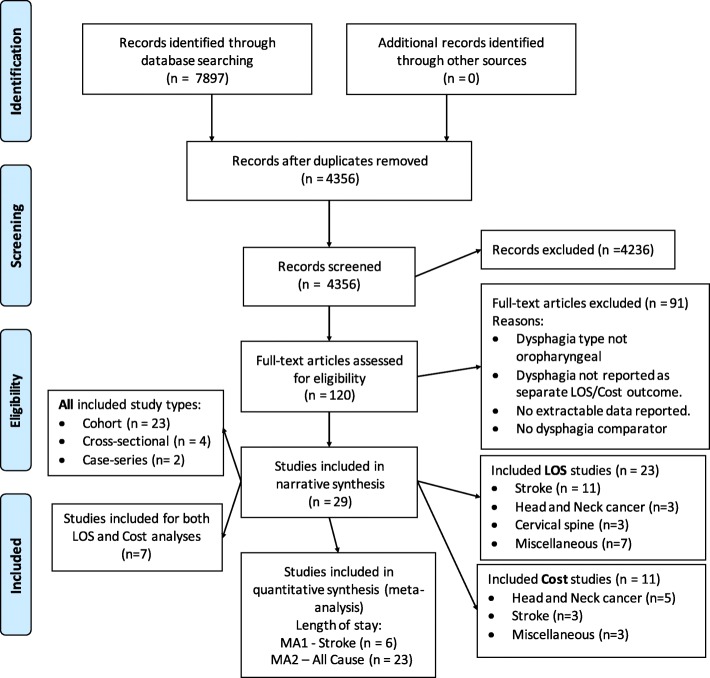
Flowchart of study identification (adapted from Moher, et al., [20])

A PRISMA flow diagram (see Fig. 1) was used to report the number of records identified by the searches, the number after deduplication, papers identified through other means, the number included after initial screening, and the papers excluded and reasons for this.

### Method of the review

#### Study selection

Identified studies were assessed for relevance to the review based on information in the title, abstract and keywords by four independent reviewers. Pairs of reviewers (SA and SD, JM and SW) independently screened half of the abstracts, and then compared and discussed those selected for inclusion and exclusion until consensus was achieved. A third reviewer was consulted if consensus could not be reached. Articles that appeared to meet the inclusion criteria were retrieved for full text review. The same pairs of reviewers independently reviewed half of these retrieved articles according to the inclusion criteria, and then compared these to determine whether they would be included for further analysis.

#### Critical appraisal and data extraction

Pairs of reviewers undertook critical appraisal of the included studies (SA and SD, JM and SW), utilising standardised and validated critical appraisal tools. Appraisal tools were specific to study design and are freely available [19].

Assessment of methodological quality focused on the appraisal of the sampling method, measurement of dysphagia as the exposure of interest, recognition of confounding variables, measurement of LOS and cost outcomes, and the statistical analysis employed. Discussion occurred until consensus was reached between the pairs of authors about the rating for each item. Subsequently, the Grading of Recommendations, Assessment, Development, and Evaluations (GRADE) method was used to give a rating of the overall quality of each study [23]. Two authors (JM and SW) assigned a GRADE rating of High, Moderate, Low or Very low after reaching consensus. As per the GRADE method, quality is considered Low for observational studies but can be upgraded one step if the effect size is large, there is a clear dose-response relationship or when confounders are fully considered. A data extraction tool was developed and piloted (by JM and SW) to extract all relevant data about LOS and cost from included studies. Whilst data were extracted from all studies that met the inclusion criteria, those judged by the authors as utilising less reliable or valid measures of oropharyngeal dysphagia, in accordance with internationally recognised best practice recommendations for the assessment of the presence of oropharyngeal dysphagia [24], were rated using the GRADE method as of lower quality for narrative analysis. Extracted data is summarised in Table 1.

**Table 1:** Methodological quality of included studies

Cohort studies											
Authors, date	Design	Sampling - Cohort selection		Presence of dysphagia			Confounders		Outcomes	Statistics	Overall quality rating
	Retrospective or Prospective	Recruited from similar population	Cohorts similar at baseline	Measure used valid and reliable	Measured similarly for correct allocation	Groups free of dysphagia at start	Identified	Approp strategies to deal with	Measure used valid and reliable	Approp stats used	
Altman et al., 2010 [11]	Retro	+	˜	x	+	˜	+	+	+	+	Moderate
Arnold et al., 2016 [27]	Retro	+	+	+	+	˜	+	+	+	+	Moderate
Bonilha, et al., 2014 [15]	Retro	+	˜	x	+	˜	+	˜	+	+	Moderate
Bradley et al., 2011 [28]	Retro	+	+	+	+	+	+	x	+	+	Low
Chaw et al., 2012 [35]	Pro	+	+	+	+	+	+	˜	+	˜	Low
Chen & Ke, 2016 [36]	Retro	+	˜	+	+	˜	+	+	+	+	Moderate
Falsetti et al., 2009 [37]	Pro	+	˜	+	+	˜	+	x	+	+	Low
Ferraris et al., (2001) [38]	Pro	+	˜	+	+	˜	+	˜	+	+	Low
Genther & Gourin, 2015 [16]	Retro	+	˜	x	+	˜	+	+	+	+	Moderate
Gourin et al., 2015 [17]	Retro	+	˜	x	+	˜	+	+	+	+	Moderate
Guyomard et al., 2009 [39]	Retro	+	˜	+	+	+	+	+	+	+	Moderate
Hogue et al., 1995 [29]	Retro	+	+	+	+	˜	+	+	+	+	Moderate
Holmes et al., 2016 [32]	Retro	+	˜	+	+	+	˜	x	+	+	Low
Macht et al., 2013 [40]	Retro	+	˜	+	+	˜	˜	˜	+	+	Low
Macht et al., 2011 [41]	Retro	+	˜	+	+	˜	+	+	+	+	Moderate
Nilsson et al., 1998 [31]	Pro	+	+	x	+	˜	x	x	+	+	Low
Odderson et al., 1995 [42]	Pro	+	+	x	+	˜	x	x	+	+	Low
Rao et al., 2005 [26]	˜	˜	˜	˜	˜	˜	x	x	+	˜	Very low
Smithard et al., 1996 [12]	Pro	+	+	+	+	˜	+	˜	+	+	Moderate
Teasell et al., 2002 [43]	Retro	+	+	+	+	˜	x	x	+	+	Low
Tian et al., 2013 [44]	Retro	+	+	x	+	˜	+	+	+	+	Moderate
Westergren et al., 1999 [33]	Pro	+	+	+	+	˜	+	x	+	+	Low
Young et al., 1990 [30]	Retro	+	+	x	+	˜	+	x	+	x	Low

Key:

+ completed and reported in study

X not completed in study

˜ unclear whether completed as not reported

#### Data analysis

Where appropriate, extracted data was combined in random effects meta-analysis, as we anticipated that the data would be highly variable [25].

Whilst all included studies reported LOS in days, the consistency of reporting was variable. No study provided information about the exact parameters that defined LOS, so for the purpose of this review, we used the AIHW definition: LOS is measured in patient days; and is the period of admitted patient care between a formal admission and formal separation [22].

Reported costs also varied widely, ranging from costs of the primary hospital admission only to total health care costs from diagnosis to end of life. Given this variation, meta-analysis of the cost findings was not considered appropriate so findings have been tabulated (Table 2) and discussed in narrative summary.

**Table 2:** Studies included in the descriptive analysis of dysphagia attributable expenditure

Authors, date	Total sample size (dysphagia)	Primary clinical diagnosis	Data source (years)	Costs inclusive of	Mean US$ difference = attributable cost of dysphagia	% difference	*P* value	Statistical test used
Cohort studies								
Bonilha, et al., 2014 [15]	2883 (317)	Ischaemic stroke	US Medicare billing records (2004–2005)	Cost over and above general care (dysphagia attributable cost)	4510	25.4	< 0.0001	Gamma distributed generalised linear model
Chen & Ke, 2016 [36]	237 (118)	Haemorrhagic stroke	National health insurance billing, Taiwan (2002–2012)	Total medical cost (insurer and co-payments by individual)	1393.7	23.5	< 0.001	Multivariate linear regression
Ferraris et al., (2001) [38]	1042 (31)	Post-cardiac surgery	Hospital surgical data (1998–1999)	Hospital related costs per admission	33,323	92.1	< 0.0001	Mann-Whitney test of inference
Genther & Gourin, 2015 [16]	61,740 (4461)	Head and Neck cancer	Nation-wide inpatient sample, Health care cost and utilisation project, Agency for Healthcare, Research and Quality (AHRQ) (2001–2010)	Total cost per admission	3976	16.4	< 0.001	Generalised linear regression
Gourin et al., 2015 [17]	2370 (616)^a^	Head and Neck cancer	National surveillance, Epidemiology and End Results (SEER)- Medicare linked database (2004–2007)	Total Medicare paid amounts	65,766^b^	102	Not reported	Multivariate linear regression
Rao et al., 2005 [26]	4551 (908)	Stroke	Not reported	Actual cost of care	5107	28.5	< 0.001	Not reported
Tian et al., 2013 [44]	8977 (485)	Alzheimer’s disease	Medicare and Marketscan Commercial databases (2006–2010)	Total health care cost (hospitalisation, outpatient, ER, pharmacy)	3620	35.2	Not reported	Multivariate linear regression
Cross-sectional								
Chan et al., 2013 [45]	7791 (467)	Head and Neck cancer	AHRQ (2001–2008)	Hospital related costs only per admission	8201	52	< 0.001	Generalised linear regression
Semenov et al., 2012 [46]	93,663 (5245)	Head and Neck cancer	AHRQ (2003–2008)	Hospital related costs only per admission	2609	12	< 0.001	Generalised linear regression
Starmer et al., 2014 [47]	1,649,871 (32,922)	Anterior cervical disc surgery	AHRQ (2001–2010)	Hospital related costs only per admission	4692	28.6	< 0.001	Generalised linear regression
Ward et al., 2012 [48]	17,281 (443)	Head and Neck cancer	AHRQ (2003–2008)	Hospital related costs only per admission	6663	28.3	< 0.001	Generalised linear regression

^a^Data visually extracted from Figure

^b^Raw data provided by authors

**Fig. 2:**
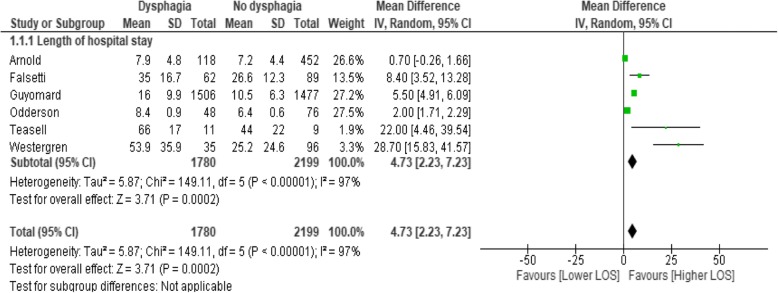
Meta-analysis of dysphagia attributable LOS data reported in cohort studies of patients presenting with stroke

**Fig. 3:**
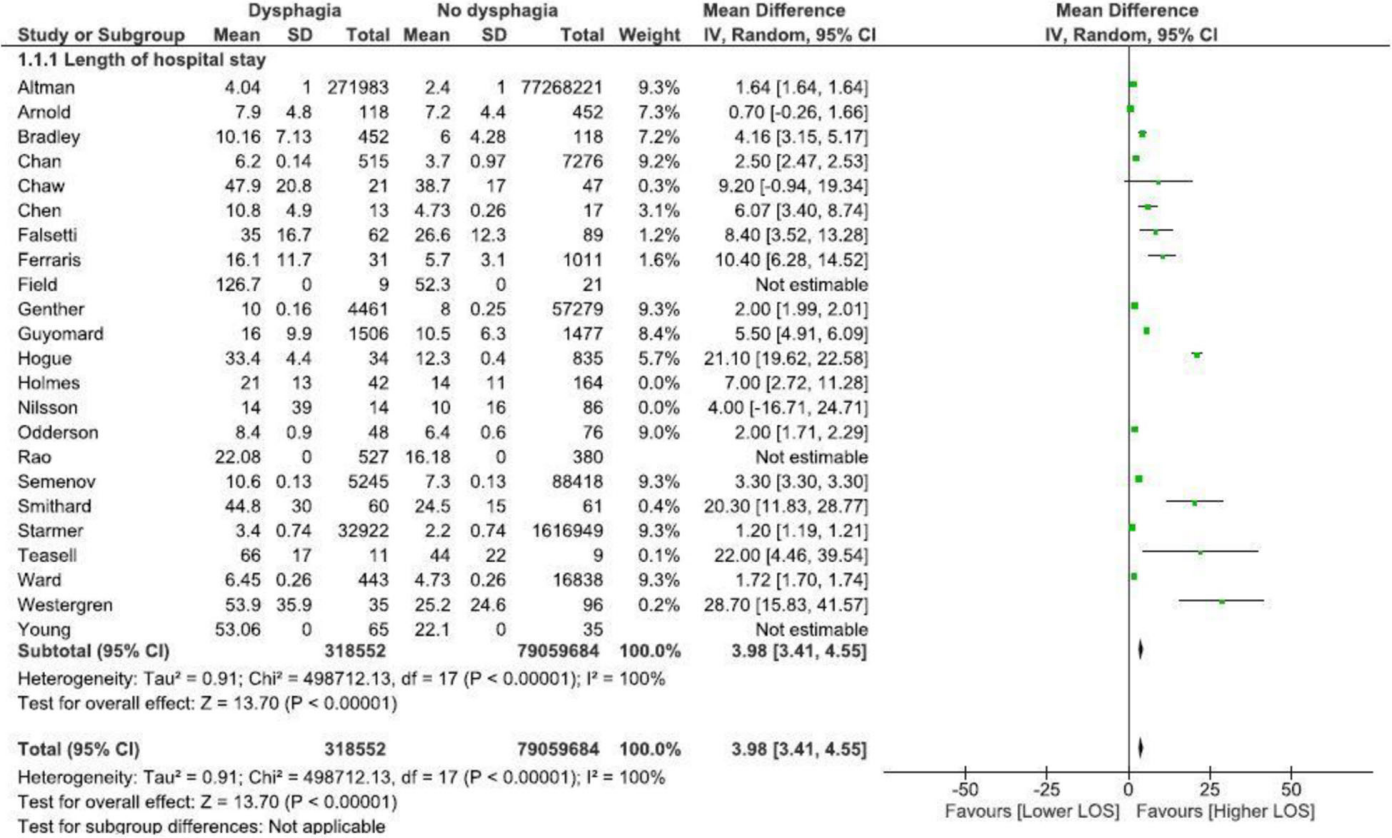
Dysphagia increases LOS, regardless of admission cause

**Fig. 4:**
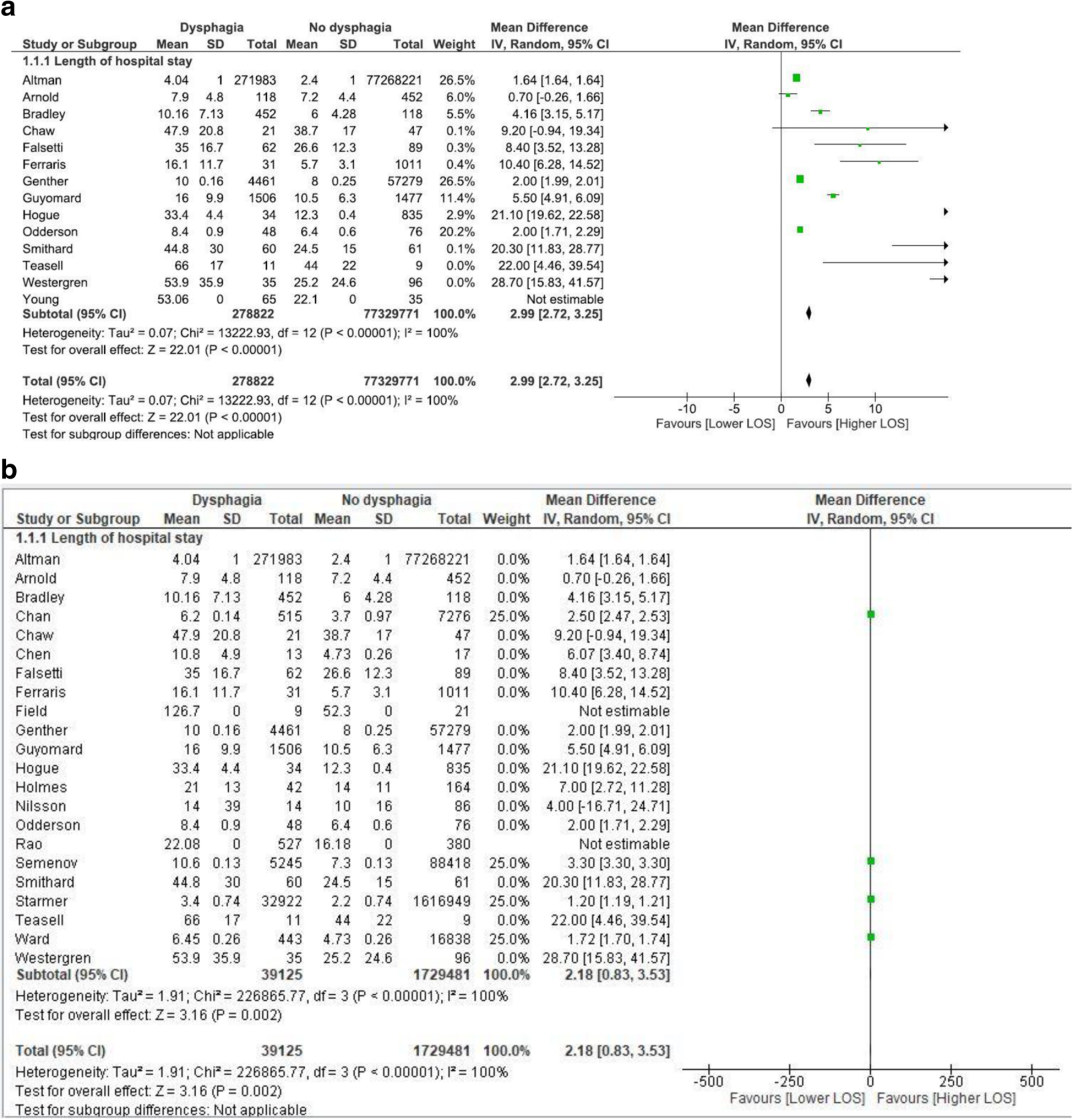
**a** Analysis of cohort studies. **b**. Analysis of cross section studies

**Fig. 5:**
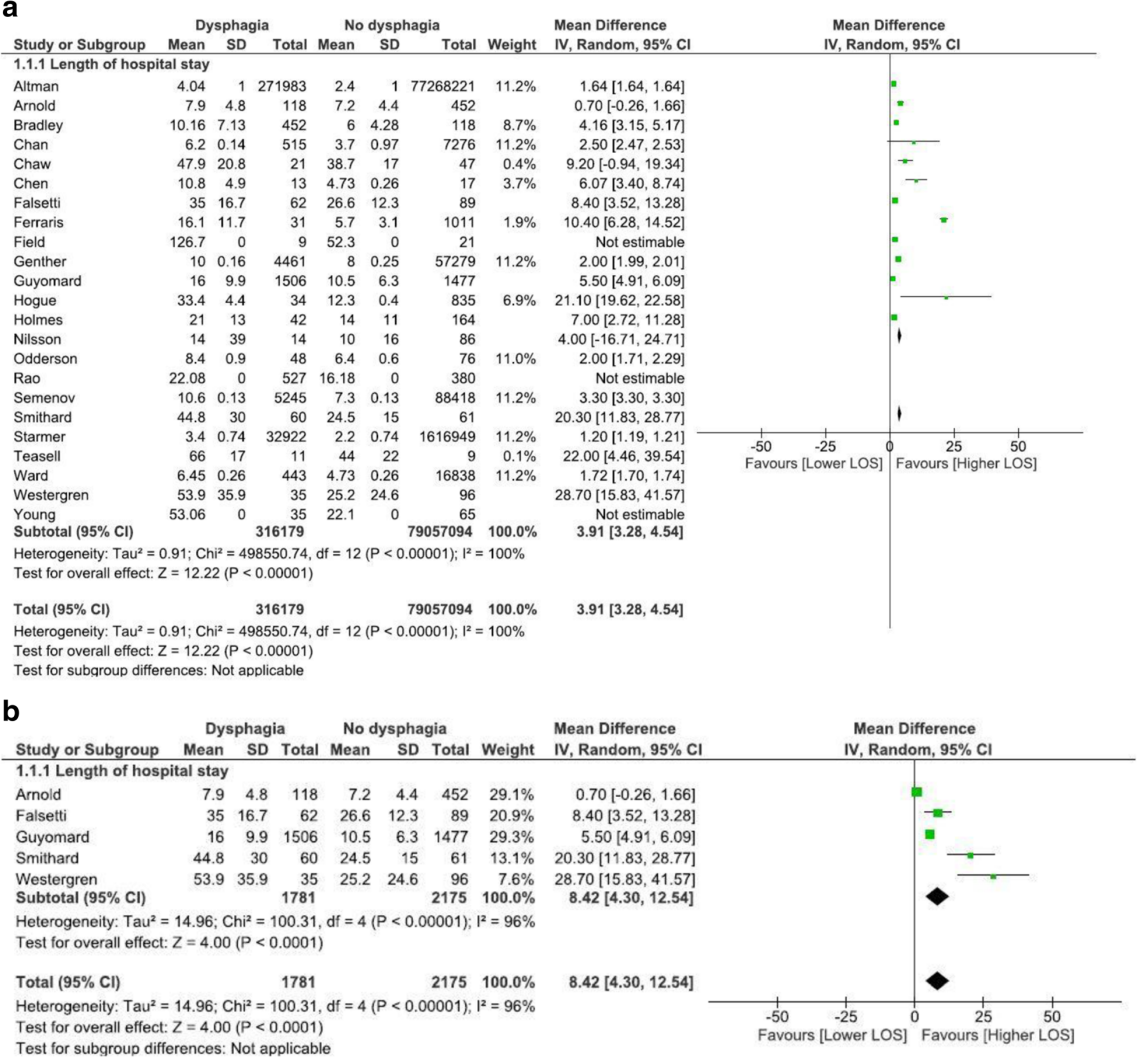
**a** Analysis by region: Northern America. **b**. Analysis by region: Europe

LOS data is presented in Table 3. We selected studies including patients with the common diagnosis of stroke for meta-analysis, as stroke-related dysphagia is commonly researched, known to increase medical acuity [12], and is the subject of standard, agreed international guidelines for acute dysphagia management [5]. As other diagnostic groups are less subject to consistent guidelines for dysphagia management, only cohort studies that sampled patients with stroke (either ischaemic or haemorrhagic) were selected for meta-analysis to determine whether dysphagia significantly contributed to an increased LOS (Table 4 and Fig. 2). As the impact for stroke was striking, and dysphagia is a symptom common to many conditions, a subsequent ‘all causes’ meta-analysis was conducted to determine whether this finding generalised to more diverse primary diagnoses and study designs. This second meta-analysis statistically combined data from all included studies that report LOS data related to oropharyngeal dysphagia regardless of study design (see Fig. 3), and then considered the impact of cohort study design (see Fig. 4a) and cross-sectional study design (see Fig. 4b) in subgroup analysis. Finally, as the included studies were prominently from either Northern America or Europe, we grouped studies together by region in further sub-group analysis (Fig. 5).

**Table 3:** Included studies reporting dysphagia attributable length of stay data

Study type	Citation	Primary clinical diagnosis	Size of population that LOS is based on	Reported LOS Dysphagia (mean unless otherwise stated)	Reported LOS No-dysphagia OR Total sample (mean unless otherwise stated)	Reported significance
cohort	Altman, K. W., Yu, G. P., & Schaefer, S. D. (2010) USA [11]	Acute hospitalisations (conditions detailed in Table 4)	Admissions = 77,540,204 Dysphagia = 271,983	median 4.04 days (4.0–5.0; 95%CI)	median 2.4 days, (3.0–3.0; 95%CI) (data error?)	not reported
cohort	Arnold, M., Liesirova, K., Broeg-Morvay, A., Meisterernst, J., Schlager, M., Mono, M. L.,. Sarikaya, H. (2016) Switerland [27]	Ischaemic stroke	No dysphagia *n* = 452; mean age 64.9 years (SD = 14); Dysphagia *n* = 118 (20.7%); mean age 65.6 years (SD 14.5).	Total hospital LOS = 7.9 (SD = 4.8 days); Stroke unit LOS = 4.4 (SD = 2.8)	Total hospital LOS 7.2 (SD = 4.4); Stroke unit LOS 2.7 (SD = 2.4)	Total hospital LOS, *p* < 0.145; Stroke unit LOS *p* < 0.001
cohort	Bradley, J. F., 3rd, Jones, M. A., Farmer, E. A., Fann, S. A., & Bynoe, R. (2011) USA [28]	Cervical spine injury (blunt trauma)	No dysphagia *n* = 19, mean age 36.9y; Dysphagia *n* = 37, mean age 42.2y	Total LOS = 10.162 days (SD = 7.13); ICU LOS = 5.486 days (SD = 6.06).	Total LOS 6 days (SD 4.28); ICU LOS 4 days (SD 8.1)	Total LOS *p* = 0.083; ICU LOS *p* = 0.019
cohort	Chaw, E., Shem, K., Castillo, K., Wong, S. L., & Chang, J. (2012) USA [35]	Tetraplegia	Admissions *n* = 63; mean age 43 years (SD = 17.2); Dysphagia *n* = 21 (30.9%); mean age 48.6y (SD = 18.4)	47.9 (+/−20.8) days	38.7 (+/−17.0) days	*p* = 0.87
cohort	Falsetti, P., Acciai, C., Palilla, R., Bosi, M., Carpinteri, F., Zingarelli, A.,. .Lenzi, L. (2009) Italy [37]	neurorehab (non acute) ischaemic or haemorrhagic stroke	No dysphagia *n* = 89 (58.9%); mean age 78.6y (SD = 6.6); Dysphagia *n* = 62 (41%); mean age 80.7y (SD = 5.4)	35 days (range = 13–93, SD = 16.7)	26.6 days (range = 6–60, SD = 12.3)	*p* = 0.0012
cohort	Ferraris, V. A., Ferraris, S. P., Moritz, D. M., & Welch, S. (2001) USA [38]	post cardiac surgery	No dysphagia *n* = 1011 (97%), mean age = 62.8 (SD = 11.8); Dysphagia *n* = 31 (3%), mean age 71.9y (SD = 8.3)	16.1 days (SD = 11.7)	5.7 days (SD = 3.1)	*p* < 0.0001
cohort	Genther, D. J., & Gourin, C. G. (2015) USA [16]	Head and neck cancer patients who underwent ablative surgery.	61,740 patients, median age 73y (range 66–104); Dysphagia *n* = 4461 (7.2%)	10 days. Intercept + 0.2242 (0.1419–0.3065 95%CI)	8 days, intercept 0.8448 (0.7211–0.9684 95%CI)	*p* < 0.001
cohort	Guyomard, V., Fulcher, R. A., Redmayne, O., Metcalf, A. K., Potter, J. F., & Myint, P. K. (2009) UK [39]	Stroke (ischaemic or haemorrhagic)	No dysphagia *n* = 1477, mean age 75y (SD = 12.1); Dysphagia *n* = 1506, Mean age 79y (SD = 9.7).	16.0 days (SD = 9.9)	10.5 days (SD = 6.3)	< 0.001
cohort	Hogue, C. W., Jr., Lappas, G. D., Creswell, L. L., Ferguson, T. B., Jr., Sample, M., Pugh, D., Lappas, D. G. (1995) USA [29]	acute hospitalisation with cardiopulomoary bypass	No dysphagia *n* = 835, mean age 63 y (+/− 0.4), Dysphagia *n* = 34, 71y (+/−2)	ICU = 15.1 days (+/− 3.1), Post operative LOS = 33.4 days (+/− 4.4)	ICU = 4.4 days (+/− 0.2); Post operative LOS = 12.3 days (+/− 0.4)	*p* = 0.0001
cohort	Holmes, S. R. M., Sabel, A. L., Gaudiani, J. L., Gudridge, T., Brinton, J. T., & Mehler, P. S. (2016) USA [32]	Anorexia nervosa	No dysphagia *n* = 164, 27 y (22–35); Dysphagia *n* = 42, 32 years (23–47)	median = 21 days (IQR = 14–27)	Median = 14 days (IQR = 9–20)	*p* < 0.001
cohort	Nilsson, H., Ekberg, O., Olsson, R., & Hindfelt, B. (1998). Sweden [31]	Stroke	No Dysphagia *n* = 58; Dysphagia *n* = 14.	median = 14 days (IQR = 8–47)	median = 10 days (IQR = 6–22)	Not significant
cohort	Odderson, I. R., Keaton, J. C., & McKenna, B. S. (1995) USA [42]	Acute non-haemorrhagic stroke	No-Dysphagia *n* = 76, age = 75.3y (+/− 1.4); Dysphagia *n* = 48; age = 75.2y (+/− 1.5)	8.4 days (+/− 0.9)	6.4 days (+/− 0.6)	*p* = 0.05
cohort	Rao, N., Brady, S., Chaudhuri, G., Ruroede, K., & Caldwell, R. 2005 [26]	Stroke	No dysphagia *n* = 380 (42%); Dysphagia *n* = 527 (58%)	22.08 days	16.18 days	*p* < 0.001
cohort	Smithard, D. G., O’Neill, P. A., Parks, C., & Morris, J. 1996, UK [12]	Stroke	No dysphagia *n* = 61; Dysphagia *n* = 60 patients	44.8 days (32–62; 95%CI)	24.5 days (18–33; CI = 95%)	*p* < 0.001
cohort	Teasell, R., Foley, N., Fisher, J., & Finestone, H, 2002 Canada [43]	Medullary stroke	No Dysphagia *n* = 9, mean age = 55y (+/− 16); Dysphagia *n* = 11 (mean; age: 57 +/− 16)	Total hospital: 66 days (+/− 17); Rehabilitation: 48 days (+/− 14)	Total hospital: 44 days (+/− 22). Rehabilitation: 24 days (+/− 17)	Total hospital *p* = 0.023, Rehabilitation *p* = 0.002
cohort	Westergren, A., Hallberg, I. R., & Ohlsson, O. (1999). Sweden [33]	Stroke	Total *n* = 96, mean age 74.3y (SD = 9.6), Dysphagia *n* = 35, mean age 74.6y (SD = 7.6)	53.9 days (+/− 35.9)	25.2 days (+/− 24.6)	*p* < 0.001
cohort	Young, E. C., & Durant-Jones, L. (1990). USA [30]	Stroke	No Dysphagia *n* = 35, mean age 61 yrs.; Dysphagia *n* = 65, mean age 75 yrs	mean LOS = 53.06; median LOS = 32	mean LOS = 22.10 days; median LOS = 15 days	*p* < 0.0002 (calculated from median)
cross section	Chan, J. Y., Li, R. J., & Gourin, C. G. (2013) USA [45]	Head and neck cancer patients who underwent ablative surgery.	Total *n* = 7791, mean age 53.4 years (range 18–89); Dysphagia = 6.6% of total	Add 2.5 days compared with whole sample (95% CI .5307–.8021, estimate .6664, *p* < 0.001).	Whole sample = 3.7 days (95% CI 0.0205–0.9909)	*p* < 0.001
cross section	Semenov, Y. R., Starmer, H. M., & Gourin, C. G. (2012). USA [46]	Head and neck cancer patients who underwent ablative surgery.	Total *n* = 93,636, Dysphagia = 5.6% of total	Add 2.8 days to total sample	7.3 days (estimate: 0.1998, 95%CI 0.1364–0.2632)	*p* < 0.001
cross section	Starmer, H. M., Riley, L. H., 3rd, Hillel, A. T., Akst, L. M., Best, S. R., & Gourin, C. G. 2004, USA [47]	Anterior Cervical Disc surgery	Total *n* = 1,659,871; Dysphagia *n* = 32,922 (2%).	Add 1.2 days to total sample, estimate: 0.558 (0.05173–0.7908)	2.2 days (estimate 0.4742 (0.4523–0.4961)	*p* < 0.001
cross section	Ward, B. K., Francis, H. W., Best, S. R., Starmer, H. M., Akst, L. M., & Gourin, C. G. (2012) USA [48]	Vagus nerve injury due to vestibular schwannoma.	Total *n* = 17,281, mean age 50.4 yrs. (range 18–97); Dysphagia *n* = 443	Add 1.72 days to total sample (0.23–0.49, 95%CI)	intercept 4.73 days	*p* = 0.001
case series	Chen, C. J., Saulle, D., Fu, K. M., Smith, J. S., & Shaffrey, C. I. (2013) USA [49]	Combined anterior-posterior cervical spine surgery	Total *n* = 30, mean age = 59.1 y (range 36–80); Dysphagia *n* = 13 (43.3%), mean age 60.7 years (SD = 7.2).	10.8 days (SD 4.9)	5.9 days (SD 2.6)	*p* = 0.004; After adjustment for age, LOS remained significantly *p* = 0.021
case series	Field, L. H., & Weiss, C. J. (1989) USA [50]	Traumatic Brain Injury	Total *n* = 30, Dysphagia *n* = 9 (30%)	126.7 days	52.3 days	None; descriptive only. No indication of variance or CIs

**Table 4:** Overview of studies included in the meta-analysis of dysphagia attributable LOS reported in cohort studies of patients presenting with stroke

Citation	Primary clinical diagnosis	Reported LOS Dysphagia. Mean +/− SD days		Reported LOS No-dysphagia.Mean +/− SD days		Mean difference (days)	Varience Standard Error (SE)	t-statistic and significance level
		n =		n =				
Arnold, M., et al. (2016) Switerland [27]	Ischaemic stroke	118	Total hospitalisation: 7.9 +/− 4.8	452	Total hospitalisation: 7.2 +/− 4.4	0.7	SE: 0.464, 95% CI - 1.6107 to 0.2107	t-statistic −1.510, DF 568, Significance level *P* = 0.1317
			Stroke unit LOS: 4.4 +/− 2.8		Stroke unit LOS: 2.7 +/− 2.4	1.7	SE 0.257, 95% CI - 2.2051 to −1.1949	t-statistic −6.610, DF 568, Significance level *P* < 0.0001
Falsetti, P., et al. (2009) Italy [37]	neurorehab (non acute) ischaemic or haemorrhagic stroke	62	35 +/− 16.7	89	26.6 +/− 12.3	8.4	SE 2.360, 95% CI -13.0634 to −3.7366	t-statistic −3.559, DF 149, Significance level *P* = 0.0005
Guyomard, V., et al. (2009) UK [39]	Stroke (ischaemic or haemorrhagic)	1506	16.0+/− 9.9	1477	10.5+/− 6.3	5.5	SE 0.304, 95% CI - 6.0970 to −4.9030	t-statistic −18.063, DF 2981, Significance level *P* < 0.0001
Odderson, I. R., et al. (1995) USA [42]	Acute non-haem CVA	48	8.4 +/− 0.9	76	*n* = 76, 6.4 (+/− 0.6)	2	SE 1.039, 95% CI - 4.0571 to 0.0571	t-statistic −1.925, DF 122, Significance level *P* = 0.0566
Teasell, R., et al. 2002 Canada [43]	medullary CVA pts. with and without dysphagia	527	mean total hospital: 66 +/−17 days	380	total hospital: 44 +/−22 days	Difference 22.0	SE 8.712, 95% CI - 40.3024 to −3.6976	t-statistic − 2.525, DF 18, Significance level *P* = 0.0212
			Rehab: 48+/−14 days		Rehab 24+/−17 days	Rehab: Difference 24.0	SEr 6.924, 95% CI - 38.5474 to −9.4526	t-statistic −3.466, DF 18, Significance level *P* = 0.002
Westergren, A., et al. (1999). Sweden [33]	stroke	60	53.9 +/−35.9	61	25.2 +/−24.6	−28.7	SE 5.533, 95% CI - 39.6481 to −17.7519	t-statistic −5.187, DF 129, Significance level *P* < 0.0001

## Results

### Description of studies

Removal of duplicates from the original searches yielded 4356 studies. After verification, 120 studies were identified as potentially eligible for inclusion. Based on the full text review, studies were excluded at this point if dysphagia type was not oropharyngeal, if oropharyngeal dysphagia was not reported as a separate LOS or cost outcome, or if a comparator was not included.

Sixty studies were critically appraised and 29 studies were included for analysis (see Fig. 1). Studies excluded following critical appraisal had no extractable data. Of the 29 included studies, 23 were cohort studies, 4 cross-sectional studies and 2 were case series. Separately, cost analysis was included in 11 studies, and LOS was included in 23 studies, but 7 studies reported both cost and LOS.

Studies sampled adults with an acquired condition who were admitted to hospital for medical or surgical management. Clinical diagnoses varied, however; 13 studies evaluated outcomes for stroke patients, five studies included patients with head and neck cancer, three studies examined patient outcomes following spinal surgery and two post-cardiac surgery. Individual studies included participants with dementia, anorexia nervosa and traumatic brain injury. The methods used to diagnose oropharyngeal dysphagia varied between studies; some utilised clinical assessment and others extracted data from ICD-9 coding. Similar variability was observed for cost outcomes, as data sources included USA Medicare billing records, USA Inpatient Health Care Cost and Utilisation Project or health insurance billing records.

### Methodological quality

#### Critical appraisal of the literature

Assessment of methodological quality is summarised in Table 1. Of the 23 included cohort studies, 15 collected data retrospectively from databases or chart reviews, 7 collected data prospectively, and one conference abstract was unclear about data collection methods [26]. All studies recruited participants from a similar, well-defined population and inclusion criteria but only 11 demonstrated adequately that their cohorts were similar at baseline. Fourteen measured oropharyngeal dysphagia with methods the authors considered as valid and reliable; through swallow screening, speech-language pathology clinical assessment or instrumental assessment [24]. The remaining cohort studies (*n* = 8) used ICD-9 coding to identify cohorts. Few studies confirmed the absence of dysphagia in participants prior to the study. Many cohort studies (*n* = 17) identified relevant confounders, including age, stroke severity, comorbidity complexity but only half of these (*n* = 9) used the appropriate statistical methods to manage these. All studies measured cost and/or length of stay outcomes in well-defined, reliable and valid ways and used appropriate statistics for primary outcomes.

As all studies included in this systematic review were observational studies, they commenced with a quality rating of Low according to GRADE. Eleven (11) of the 23 cohort studies were upgraded to Moderate following critical appraisal, as they had relatively large sample sizes and considered confounders in the statistical analysis. The remainder of the cohort studies (× 12) along with the cross-sectional studies (× 4) and the case series (× 2) were rated as Low quality. No papers were excluded on the basis of methodological quality.

### Findings of the review

#### Dysphagia attributable costs

In total, seven cohort studies and four cross-sectional studies with a range of clinical diagnoses, including stroke, head and neck cancer and post-surgical care, were included for descriptive analysis of cost as an outcome (Table 2). Across these eleven studies, including three longitudinal studies, data were reported for the billing years 1998–2012 and included a total sample of 1,850,406 individuals of whom 46,013 presented with dysphagia (2.49%). Ten studies were conducted in the USA, one in Taiwan, and all reported in $USD. Settings included surgical wards (*n* = 5), acute care (*n* = 3) and inpatient rehabilitation (*n* = 1). The mean attributable cost of dysphagia across all eleven studies was USD$12,715, representing an increase in dysphagia-related expenditure compared to the relevant non-dysphagic comparator groups of 40.36%. The difference in cost for patients with oropharyngeal dysphagia compared to those without was reported as significant in nine of the eleven studies, and not reported in the remaining two studies.

#### Impact of dysphagia on LOS

In total, seventeen cohort studies, four cross-sectional studies and two case series were included that were conducted in a range of countries, most prominently the USA (15), followed by the United Kingdom (2) and Sweden (2), and single studies each from Switzerland, Italy and Canada. One conference abstract did not report the country of origin [26]. LOS data varied across the studies, which was unsurprising as the studies related to different research foci, and included patient groups with a range of clinical diagnoses (Table 3). Across these studies, data were collected from 79,378,058 individuals, including 318,506 (0.4%) with dysphagia.

Of the 23 studies, four reported median LOS, and 14 either explicitly reported mean LOS, or reported statistical methods that required a mean value. For the remaining five studies that did not report a method of LOS calculation, we have assumed a mean value, as this was supported by contextual information included in the published report.

As shown in Table 3, the 23 included studies reported on 26 admission settings, as three studies analyzed LOS for two separate settings [27–29], for example, LOS was reported for stroke unit, intensive care unit and general ward admissions. Total LOS was utilised for these studies. Dysphagia related LOS was significantly longer in 21 of the 26 reported analyses for *p* ≤ 0.05. Of the remaining five settings, three were not statistically significant, and in two the *p* value was not reported.

### Meta-analysis of LOS data for patients admitted for stroke, with and without dysphagia

There were ten studies that commonly included LOS data for patients with stroke. However, only cohort studies that sampled patients with stroke were selected for meta-analysis to reduce variation. The sample size and variance of the six studies that reported sufficient data for statistical combination were varied (as indicated by the confidence intervals (CIs) and I^2^ value) (Table 4; Fig. 2). All six studies showed an increased LOS for individuals admitted for stroke if they also had dysphagia. In all except one study [26] the increased LOS was statistically significant. Overall, based on six cohort studies and a sample of 3879 individuals, dysphagia added almost five extra days in hospital (a mean of 4.73 days more, 95% CI: 2.23, 7.23).

### Meta-analysis of LOS data for ‘all-cause’ admissions

LOS data from eighteen included studies were subsequently pooled regardless of diagnostic grouping or study design, to provide a broad overview about the relative impact of dysphagia on LOS. The meta-analysis in Fig. 3 highlights considerable variance within some of the included studies (as indicated by the CI’s), however, all studies reported an increased LOS for individuals with dysphagia. Data from three studies [26, 27, 30] did not contribute to this meta-analysis as they reported only mean LOS without standard deviation or standard error of the mean. Two studies [31, 32] that reported median and interquartile range values were also not included in the meta-analysis. Overall, based on eighteen studies and a total sample of 79,377,199 individuals, patients with dysphagia, who were 0.4% of the sample, added approximately four extra days in hospital (a mean of 3.98 days longer, 95% CI: 3.41, 4.55) compared to individuals with no dysphagia (Fig. 3). This pooled data included the studies in the stroke meta-analysis that comprised 25.9% of the sample. However, meta-analysis of ‘all cause’ studies that excluded these stroke studies also identified that the presence of dysphagia increased LOS (a mean of 4.27 days longer, 95% CI: 3.6, 4.93).

### Meta-analysis of LOS data for ‘all-cause’ admissions: Impact of study design

To investigate the variance in the data, impact of study design was considered in sub-group analysis. Dysphagia contributed an additional three days to LOS (2.99 days; 2.72, 3.25) when the data from thirteen cohort studies, representing 77,608,593 participants was combined in meta-analysis (Fig. 4a). Dysphagia contributed an additional two days to LOS (2.18 days: 95% CI: 0.83, 3.53) when data from four cross sectional studies, representing 176,806 participants was combined in meta-analysis (Fig. 4b).

### Meta-analysis of LOS data for ‘all-cause’ admissions: impact of geographical region

As studies from Northern America were prominent in the data, sub-group analysis of these compared with European studies was conducted to determine if regional differences existed. Of the twenty three included studies, sixteen were conducted in North America. Data from thirteen of these studies (79,373,273 participants) was combined in meta-analysis (see Fig. 5). Dysphagia added four days to LOS (3.91 days; 95% CI: 3.28, 4.54). Seven studies were conducted in Europe. Data from five of these studies (3958 participants) were combined in meta-analysis (see Fig. 5b). Dysphagia added eight days to LOS (8.42 days; 95% CI: 4.30, 12.54).

## Discussion

This systematic review evaluated the impact of oropharyngeal dysphagia on healthcare expenditure and patient LOS. Although varying in magnitude, overall expenditure measured via monetary cost increased by 40.36% in patients with oropharyngeal dysphagia compared to their non-dysphagic counterparts, a finding that was consistent across years and underlying clinical presentations. Analogously, the presence of oropharyngeal dysphagia added between two and eight extra days to hospital LOS, regardless of reason for admission, study design utilised, or whether the region in which the study was conducted was Northern America or Europe.

### Quality of the reviewed literature

Critical appraisal of the included studies revealed several methodological constraints that warrant discussion, and which limit the interpretation of the findings of this review. Overall, nearly half of the included cohort studies (11/23) were graded as moderate quality based on robust critical appraisal, consideration of confounders in the analysis and relatively large sample sizes. The remaining cohort studies (12/23), as well as four cross-sectional studies and two case series were graded as low quality. Thus, the overall evidence captured within this systematic review is not considered strong. This lack of strongly ranked studies may be partly attributed to the type of research questions posed, as evaluating healthcare costs and LOS are observational analyses by their nature. Therefore, prospective cohort studies with pre-identified confounders that are appropriately managed statistically are perhaps the most appropriate design, even though these are conventionally regarded as “moderate” with respect to the quality of evidence. It is unlikely that RCTs, universally considered to produce a higher quality of evidence, would be specifically designed to evaluate expenditure and LOS, although future RCTs may now add these variables as outcomes where appropriate.

The approaches to identify the presence or absence of oropharyngeal dysphagia varied across the included studies; 15/23 cohort studies assessed oropharyngeal dysphagia using direct clinical assessment, either through dysphagia screening, speech pathology clinical assessment or instrumental assessment. Different assessment processes were implemented, including factors that impeded study validity such as transparency of process, timing and staff training for dysphagia screening as well as timing and processes for speech pathology clinical assessment [31, 33]. The remaining eight cohort studies derived data from ICD-9 codes that relied on the correctness of administrative coding for oropharyngeal dysphagia at discharge, compared with directly confirming the presence of dysphagia. Data sources for these studies also varied substantially, including insurer datasets consulted retrospectively, and summarised data of hospital-incurred expenses collected prospectively. The potential for coding errors or omissions that influence entry and maintenance of these data sources may have confounded the outcomes of the reviewed studies by under-estimating the frequency of oropharyngeal dysphagia [18] and therefore the factors related to healthcare utilisation that were reported.

### Healthcare costs related to oropharyngeal dysphagia

Whilst all included studies reported cost in $USD, the studies included for cost analysis varied in the clinical populations, contexts and time points for cost measurement. These ranged from costs incurred during hospital admission, to costs from diagnosis of a condition until death. For these reasons, meta-analysis of cost data was not conducted. Despite these potential confounders and sources of variability, results from narrative analysis of the included studies indicated that patients presenting with oropharyngeal dysphagia incurred 40.36% greater costs than those without dysphagia. This compares favourably with the 42% increase identified in Patel and colleagues’ [18] USA study of patients with dysphagia of oropharyngeal and esophageal origin, despite differences in the population, underlying condition, year or country of origin of the studies. Several studies included discussion about oropharyngeal dysphagia-related expenditure, which was attributed to i. often repeated, diagnostic procedures such as videofluoroscopic evaluations of swallowing and chest x-rays, ii. management of complications of oropharyngeal dysphagia, such as malnutrition or pneumonia, iii. increased multi-disciplinary involvement over a longer period of stay in hospital, and iv. use of consumables such as enteral feeding or modified food and fluids. The finding that presence of oropharyngeal dysphagia resulted in increased expenditure regardless of diagnosis highlights the often under-recognised magnitude of this patient-related factor on healthcare systems and resources. This underscores the need for research on robust assessment, treatment approaches and practice guidelines that are more inclusive of the range of conditions that result in oropharyngeal dysphagia.

### LOS related to oropharyngeal dysphagia

It is likely that this increased expenditure is, at least in part, associated with the increased LOS related to oropharyngeal dysphagia that was a consistent finding of the included studies. Each of the seven included studies that reported data for both cost and LOS for patients with oropharyngeal dysphagia reported significant differences for both variables compared with patients without dysphagia. Whilst measures of LOS do not reflect the intensity of care or organisational processes that contribute to care [3], these studies suggest that the presence of oropharyngeal dysphagia increases hospital expenditure and utilisation across the clinical populations studied.

Initial meta-analysis was conducted about the impact of stroke-related dysphagia on LOS, which provided data to support the broad implementation of existing stroke practice guidelines for hospital based assessment and intervention practices for stroke-related dysphagia [5]. This stroke specific meta-analysis demonstrated that oropharyngeal dysphagia increased LOS by 4.73 days, extending information about the known impact of stroke-related dysphagia on patient outcomes and healthcare utilisation [13, 34]. However, this review was unable to determine whether the implementation of stroke guidelines influenced LOS, as included studies rarely reported their dysphagia procedures in accordance with these guidelines. As uptake of stroke guidelines become more commonly embedded in hospital procedures, future research should also identify whether their implementation influence measures of hospital expenditure.

The significant finding of the stroke data meta-analysis informed the decision to conduct a meta-analysis that was inclusive of all causes. Oropharyngeal dysphagia is a common patient-related factor of many conditions that is associated with greater medical acuity, and is subject to complex organisational procedures to reduce adverse sequelae, coordinate multi-disciplinary team interventions, and manage discharge outcomes. However, as data about how dysphagia independently contributes to LOS across conditions have not been reported, it is currently difficult to substantiate resources to specifically manage oropharyngeal dysphagia. In this study, meta-analysis of the ‘all-cause’ data indicated that the presence of oropharyngeal dysphagia increased LOS by four days, and this was maintained when data from the stroke meta-analysis was removed. In the ‘all cause’ cohort studies that were less variable, oropharyngeal dysphagia contributed an additional three days. The results of this ‘all-cause’ meta-analysis suggest that oropharyngeal dysphagia is a factor that increases LOS independently of underlying clinical diagnoses, adding valuable specific information for planning and resourcing of hospital services. However, this result differed substantially from Patel and colleagues’ [18] finding that dysphagia increased mean LOS by 8.8 days. Patel and colleagues combined oropharyngeal and esophageal dysphagia data from administrative coding that is likely to underestimate subclinical or less severe dysphagia presentation, and may also be influenced by contextual USA hospital admission practices where the study was conducted. These factors may have influenced the disparity in LOS reported, compared with the current study, but further research is needed to elucidate these differences.

Both patient-related and organisation-related factors are known to contribute to healthcare utilisation and resources [3]. The results of this systematic review suggest that oropharyngeal dysphagia, which is a patient-related factor subject to varied organisation-related management procedures, increases both LOS and cost across a range of clinical conditions. Further, critical appraisal highlighted the varied nature of procedures for dysphagia identification and management reported in the included studies. The results therefore provide much needed information about the independent impact of oropharyngeal dysphagia to support the development of clear guidelines and procedures to optimise and resource clinical pathways. Implementing these may reduce healthcare utilisation through reducing the adverse patient outcomes and medical management associated with oropharyngeal dysphagia. Strategies identified for specific clinical populations, such as stroke, that may have application within broader patient contexts include:

i. Implementation of validated dysphagia screening tools to allow the early detection of dysphagia.
ii. Early implementation of dysphagia management in line with current practice for acute stroke in several countries, including the USA, United Kingdom, Canada and Australia, with the aim of minimising other associated negative health outcomes such as dehydration and malnutrition as well as aspiration pneumonia.
iii. Recognition of dysphagia as a quality indicator with regular auditing and benchmarking of implementation of screening, management and patient outcomes.

## Limitations

As with all systematic reviews, the current study was limited to the inclusion of publically available sources. Whilst the search strategy included both published and grey literature, the potential for publication bias remains as no grey literature was identified. The review also intended to include studies about dysphagia-related healthcare utilisation from the perspectives of both patients and healthcare providers. However, no studies with information about independent patient costs were identified. This reveals an important gap in our understanding, as oropharyngeal dysphagia is often chronic in its presentation, and dysphagia-related costs to individuals that extend beyond their admission to hospital are likely to be substantial. Similarly, the review sought a global viewpoint of dysphagia-related healthcare utilisation, but the studies identified were prominently from Northern America and Europe. Subgroup analysis indicated substantial regional LOS variation between these two groups that perhaps reflect differences in approaches and systems for healthcare. However, the very small proportion of included studies that were not from Northern America also suggest that more research from a broader range of countries and service settings is needed to inform the development of guidelines and treatment approaches for dysphagia that are genuinely transferable to global healthcare contexts. Additionally, as only a single study was included for cost analysis that was not derived from USA data, generalizable conclusions cannot be drawn about LOS or cost.

## Conclusions

In this systematic review, we highlight the significant impact of oropharyngeal dysphagia, a symptom of many complex medical conditions, on healthcare expenditure. While there are limitations with regard to the quality of the existing literature, this review demonstrates that the presence of oropharyngeal dysphagia significantly increases both cost and LOS. This highlights the need to recognise oropharyngeal dysphagia as an important contributor to pressure on current healthcare systems. Organisational strategies that facilitate the early identification, timely and evidence-based management of oropharyngeal dysphagia across any clinical population will likely result in significant reductions in dysphagia-related negative health outcomes, and consequently LOS and attributable healthcare expenditure.

## Additional file

Additional file 1: Search terms. (DOCX 20 kb)

## Abbreviations

AIHW: Australian Institute of Health and Welfare
CI: Confidence interval
CINAHL: Cumulative index to nursing and allied health literature
DRG: Diagnosis-related group
GRADE: Grading of recommendations, assessment, development, and evaluations
ICD: International classification of disease
LOS: Length of stay
PDF: Portable document format
PRISMA: Preferred Reporting Items for Systematic Reviews and Meta-analysis
RCTs: Randomised controlled trials
USA: United States of America
USD: United States Dollars
