# Supplementary material for: Impact of oropharyngeal dysphagia on healthcare cost and length of stay in hospital: a systematic review
Source: BMC Health Serv Res. 2018 Aug 2;18:594. doi: 10.1186/s12913-018-3376-3 (PMC6090960; doi:10.1186/s12913-018-3376-3)
Supplement: Supplementary file 1 — Search terms. (DOCX 20 kb) [file 12913_2018_3376_MOESM1_ESM.docx]

**Additional file 1**

## Medline

AAAdysphagia

#
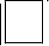
Run 29/2/16 Search Strategy:

| **#** | **Searches** | **Results** |
| --- | --- | --- |
| 1 | (aphagi* OR dysphagi* OR aglutition OR swallow* OR deglutition disorder*).tw. | 38283 |
| 2 | deglutition disorders/ | 16264 |
| 3 | 1 OR 2 | 43847 |
| 4 | exp Insurance, Health/ OR medical assistance/ OR medicaid/ OR medicare/ OR exp Insurance, Health/ | 139385 |
| 5 | (cost* OR "medical assistance" OR medicaid OR financ* OR expense* OR econom* OR expenditure* OR price* OR pricing OR money OR budget* OR funds OR insurance).tw. | 750156 |
| 6 | economics/ OR quality-adjusted life years/ OR "costs and cost analysis"/ OR cost-benefit analysis/ OR health care costs/ OR cost allocation/ OR hospital costs/ OR cost control/ OR cost of illness/ OR health expenditures/ OR budgets/ OR financial management/ | 223229 |
| 7 | ((Short OR brief OR length OR long) adj3 (stay* OR hospital* OR admission*)).tw. | 63691 |
| 8 | "Length of Stay"/ | 65057 |
| 9 | 4 OR 5 OR 6 OR 7 OR 8 | 990714 |
| 10 | 3 and 9 | 1888 |
| 11 | ANIMALS/ not (HUMANS/ and ANIMALS/) | 4156964 |
| 12 | 10 not 11 | 1817 |
| 13 | limit 12 to english language | 1636 |

## Cinahl

Run 23/2/16

Saved AAAdeglutition

| **#** | **Query** | **Limiters/Expanders** | **Results** |
| --- | --- | --- | --- |
| S1 | (MH "Deglutition Disorders") OR (MH "Swallowing Therapy") | Search modes - Boolean/Phrase | 4,265 |
| S2 | TI ( (aphagi* OR dysphagi* OR aglutition OR swallow* OR “deglutition disorder*”) ) OR AB ( (aphagi* OR dysphagi* OR aglutition OR swallow* OR “deglutition disorder*”) ) | Search modes - Boolean/Phrase | 6,008 |
| S3 | S1 OR S2 | Search modes - Boolean/Phrase | 7,296 |
| S4 | (MH "Economics") OR (MH "Costs and Cost Analysis") OR (MH "Cost Control") OR (MH "Health Care Costs") OR (MH "Nursing Costs") | Search modes - Boolean/Phrase | 42,918 |
| S5 | (MH "Cost Benefit Analysis") OR MH "Quality- Adjusted Life Years") OR (MH "Economic Aspects of Illness") OR (MH "Health Facility Costs") | Search modes - Boolean/Phrase | 21,408 |
| S6 | (MH "Insurance, Health") OR (MH "Medicaid") OR (MH "Medicare") | Search modes - Boolean/Phrase | 46,359 |
| S7 | TI ( ( (Short OR brief OR length OR long) N3 (stay* OR hospital* OR admission*) ) ) OR AB ( ( (Short OR brief OR length OR long) N3 (stay* OR hospital* OR admission*) ) ) | Search modes - Boolean/Phrase | 15,575 |
| S8 | TI ( ( (cost* OR "medical assistance" OR medicaid OR financ* OR expense* OR econom* OR expenditure* OR price* OR pricing OR money OR budget* OR funds OR insurance) ) ) OR AB ( ( (cost* OR "medical assistance" OR medicaid OR financ* OR expense* OR econom* OR expenditure* OR price* OR pricing OR money OR budget* OR funds OR insurance) ) ) | Search modes - Boolean/Phrase | 150,775 |
| S9 | (MH "Length of Stay") | Search modes - Boolean/Phrase | 18,210 |
| S10 | S4 OR S5 OR S6 OR S7 OR S8 OR S9 | Search modes - Boolean/Phrase | 226,061 |
| S11 | S3 AND S10 | Search modes - Boolean/Phrase | 443 |
| S12 | S3 AND S10 | Narrow by Language:  - english Search modes - Boolean/Phrase | 429 |

## PsycInfo


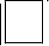
Search Strategy: Run : 29/2/16

| **#** | **Searches** | **Results** |
| --- | --- | --- |
| 1 | Dysphagia/ | 641 |
| 2 | (aphagi* OR dysphagi* OR aglutition OR swallow* OR deglutition disorder*).tw. | 3613 |
| 3 | 1 OR 2 | 3619 |
| 4 | Economics/ OR health care costs/ OR budgets/ OR "Costs and Cost Analysis"/ OR money/ OR finance/ OR funding/ | 46392 |
| 5 | (cost* OR medical assistance OR medicaid OR financ* OR expense* OR econom* OR expenditure* OR price* OR pricing OR money OR budget* OR funds OR insurance).tw. | 253768 |
| 6 | health insurance/ OR medicaid/ OR medicare/ | 6658 |
| 7 | ((Short OR brief OR length OR long) adj3 (stay* OR hospital* OR admission*)).tw. | 9333 |
| 8 | Treatment Duration/ | 3533 |
| 9 | 4 OR 5 OR 6 OR 7 OR 8 | 268285 |
| 10 | 3 and 9 | 188 |
| 11 | ANIMALS/ not (HUMANS/ and ANIMALS/) | 6292 |
| 12 | 10 not 11 | 188 |
| 13 | limit 12 to english language | 179 |

**Scopus**

Run 10/3/16 N = 2904

( TITLE-ABS-KEY ( aphagi* OR dysphagi* OR aglutition OR swallow* OR "deglutition disorder*" ) AND SUBJAREA ( mult OR medi OR nurs OR vete OR dent OR heal ) ) AND ( ( TITLE-ABS-KEY ( ( cost* OR "medical assistance" OR medicaid OR financ* OR expense* OR econom* OR expenditure* OR price* OR pricing OR money OR budget* OR funds OR insurance ) ) OR TITLE-ABS-KEY ( ( short OR brief OR length OR long ) W/3 ( stay* OR hospital* OR admission* )

) ) AND SUBJAREA ( mult OR medi OR nurs OR vete OR dent OR heal ) ) AND ( LIMIT-TO ( DOCTYPE , "ar" ) OR LIMIT-TO ( DOCTYPE , "re" ) OR LIMIT-TO ( DOCTYPE , "cp" ) ) AND ( LIMIT- TO ( LANGUAGE , "English" ) )

## Pubmed

N=268

Run 10/3

Search (((((aphagi*[Title/Abstract] OR dysphagi*[Title/Abstract] OR aglutition[Title/Abstract] OR swallow[Title/Abstract] OR swallowing[Title/Abstract] OR deglutition disorder[Title/Abstract] OR deglutition disorders[Title/Abstract]))) AND ((((cost[Title/Abstract] OR costings[Title/Abstract] OR costs[Title/Abstract] OR costing[Title/Abstract] OR "medical assistance"[Title/Abstract] OR medicaid[Title/Abstract] OR finance[Title/Abstract] OR finances[Title/Abstract] OR expense[Title/Abstract] OR expenses[Title/Abstract] OR economic[Title/Abstract] OR economics[Title/Abstract] OR expenditure[Title/Abstract] OR expenditures[Title/Abstract] OR price[Title/Abstract] OR prices[Title/Abstract] OR pricing[Title/Abstract] OR money[Title/Abstract] OR budget[Title/Abstract] OR budgets[Title/Abstract] OR budgeting[Title/Abstract] OR funds[Title/Abstract] OR insurance[Title/Abstract]))) OR ((((stay[Title/Abstract] OR stays[Title/Abstract] OR hospital[Title/Abstract] OR hospitals[Title/Abstract] OR admission[Title/Abstract] OR admissions[Title/Abstract]))) AND ((Short[Title/Abstract] OR brief[Title/Abstract] OR length[Title/Abstract] OR long[Title/Abstract])))))) NOT medline[sb]

## Cochrane

Run 10/3

(aphagi* OR dysphagi* OR aglutition OR swallow* OR “deglutition disorder*”) AND ((cost* OR "medical assistance" OR medicaid OR financ* OR expense* OR econom* OR expenditure* OR price* OR pricing OR money OR budget* OR funds OR insurance) OR ((Short OR brief OR length OR long) NEAR/3 (stay* OR hospital* OR admission*)))

## Cochrane Database of Systematic Reviews : Issue 3 of 12, March 2016

N=43

## Database of Abstracts of Reviews of Effect : Issue 2 of 4, April 2015

N=3

## Cochrane Central Register of Controlled Trials : Issue 2 of 12, February 2016

N=227

## NHS Economic Evaluation Database : Issue 2 of 4, April 2015

N=19

## Health Technology Assessment Database : Issue 1 of 4, January 2016

N=1

## Proquest

Run 20/3

Limited to Scholarly Journals

N=633

Database dedups, so 581 sent to Endnote Ti, Ab, Sh

ti((aphagi* OR dysphagia* OR deglutition OR swallow* OR "deglutition disorder*") AND ((cost* OR "medical assistance" OR medicaid OR finance* OR expense* OR economy* OR expenditure* OR price* OR pricing OR money OR budget* OR funds OR insurance) OR ((Short OR brief OR length OR long) NEAR/3 (stay* OR hospital* OR admission*)))) OR ab((aphagi* OR dysphagia* OR deglutition OR swallow* OR "deglutition disorder*") AND ((cost* OR "medical assistance" OR medicaid OR finance* OR expense* OR economy* OR expenditure* OR price* OR pricing OR money OR budget* OR funds OR insurance) OR ((Short OR brief OR length OR long) NEAR/3 (stay* OR hospital* OR admission*)))) OR su((aphagi* OR dysphagia* OR deglutition OR swallow* OR "deglutition disorder*") AND ((cost* OR "medical assistance" OR medicaid OR finance* OR expense* OR economy* OR expenditure* OR price* OR pricing OR money OR budget* OR funds OR insurance) OR ((Short OR brief OR length OR long) NEAR/3 (stay* OR hospital* OR admission*))))

## Web of Science

20/3

N=1607

(aphagi* OR dysphagi* OR aglutition OR swallow* OR “deglutition disorder*”) AND ((cost* OR "medical assistance" OR medicaid OR financ* OR expense* OR econom* OR expenditure* OR price* OR pricing OR money OR budget* OR funds OR insurance) OR ((Short OR brief OR length OR long) NEAR/3 (stay* OR hospital* OR admission*)))

You searched for: TOPIC: ((aphagi* OR dysphagi* OR aglutition OR swallow* OR “deglutition disorder*”) AND ((cost* OR "medical assistance" OR medicaid OR financ* OR expense* OR econom* OR expenditure* OR price* OR pricing OR money OR budget* OR funds OR insurance) OR ((Short OR brief OR length OR long) NEAR/3 (stay* OR hospital* OR admission*))))

Refined by: LANGUAGES: ( ENGLISH ) AND [excluding] WEB OF SCIENCE CATEGORIES: ( ECOLOGY OR ZOOLOGY OR CELL BIOLOGY OR BIOLOGY OR EVOLUTIONARY BIOLOGY OR VETERINARY SCIENCES OR PARASITOLOGY OR OCEANOGRAPHY OR BIODIVERSITY CONSERVATION OR FISHERIES OR GEOSCIENCES MULTIDISCIPLINARY OR AGRICULTURE DAIRY ANIMAL SCIENCE OR WATER RESOURCES OR BIOPHYSICS OR MARINE FRESHWATER BIOLOGY OR ENGINEERING BIOMEDICAL OR MATHEMATICAL COMPUTATIONAL BIOLOGY OR BIOTECHNOLOGY APPLIED MICROBIOLOGY OR GEOGRAPHY PHYSICAL OR ENGINEERING GEOLOGICAL OR ENGINEERING CIVIL )

Timespan: All years. Indexes: SCI-EXPANDED, SSCI, A&HCI, CPCI-S, CPCI-SSH, ESCI, CCR-EXPANDED, IC.
